# The intrinsic value of self-organized sport and physical activity: a narrative review

**DOI:** 10.3389/fspor.2026.1911210

**Published:** 2026-09-22

**Authors:** Sindre Herskedal Fosstveit

**Affiliations:** 1 Department of Sport Science and Physical Education, Faculty of Health and Sport Sciences, University of Agder, Kristiansand, Norway; 2 Tverga, Oslo, Norway

**Keywords:** autonomy, informal sport, intrinsic value, lifestyle sport, physical activity promotion, self-determination theory, self-organized physical activity, sport policy

## Abstract

In many populations, much of people's physical activity takes place outside clubs and organized competition, in self-organized forms that participants run themselves, from walking and strength training to skateboarding, parkour, and climbing. Policy and research tend to treat these forms as a residual category and to value them, when at all, for the same instrumental outcomes used to justify organized sport. This narrative review takes a different starting point and asks what is distinctively valuable about physical activity being self-organized. Its central argument is that the worth of these forms is bound up with the self-organizing itself. Because participants govern what they do, the activity directly supports autonomy, the basic psychological need most directly afforded by self-organizing, while mastery on one's own terms, self-expression and identity, meaning, and belonging are supported more variably. These qualities give the activity its intrinsic value, the worth it has as it is experienced, as distinct from the utility value used to justify public spending on it. Self-organization affords these qualities without guaranteeing them; whether they are realized depends on the character of the setting, the social relations within it, and the participant's access and social position. Autonomy and the immediate rewards of moving characterize the category broadly, while identity, meaning, and belonging are most fully realized in its social, scene-based forms. Drawing on research in physical activity, self-determination theory, the sociology of sport, and the philosophy of sport, the review synthesizes the evidence for each quality and distinguishes intrinsic value from intrinsic motivation. It then sets out why this value matters beyond the individual: self-organized physical activity is widespread, reaches people the organized system does not, and supports the motivation associated with lifelong participation. Evidence that qualifies the account is also weighed, including findings that participation reflects existing social position and that institutionalizing self-organized settings can erode their value. Recognizing this intrinsic value has implications for how the activity is provided for, measured, and prioritized, including provision that preserves autonomy rather than eroding it through formalization.

## Introduction

Physical activity is among the most consequential of everyday health behaviors, and physical inactivity is a leading risk factor for noncommunicable disease and premature mortality worldwide (1). The dominant policy response has been to promote physical activity for the health, social, and economic returns it produces, and current guidance counts physical activity in any domain, from sport and leisure to walking, cycling, and household tasks (2). Much of this physical activity is self-organized. People walk, run, lift, ride, climb, skate, and play outside clubs and organized competition, deciding for themselves what they do, when, with whom, and to what end. In what follows, organized sport refers to physical activity provided through membership-based clubs, with coaches, fixed times, and competitive formats. Self-organized sport and physical activity, a category that includes forms studied as informal, lifestyle, and action sport, refers to leisure-time physical activity that participants initiate and govern themselves, without such structures. Physical activity in general is broader still: bodily movement in any domain of daily life, whatever its organization. This review concerns leisure-time sport and exercise of this self-organized kind; physical activity that is incidental to other purposes, such as transport or household work, lies outside its scope, as does curriculum-based physical education, which belongs to compulsory schooling, not to voluntary leisure. In population surveys, the settings where people are most often active are informal ones such as parks, outdoor spaces, and the home, rather than sports clubs (3). The institutions of sport, however, are built around a different model. Funding, facilities, and research follow the club and federation structure that has long defined the European sport landscape, a model that captures only part of how people are actually active (4, 5). Within this system, self-organized physical activity occupies a residual position. It is recorded as what remains once organized participation has been counted, and valued, where it is noticed at all, for the same instrumental outcomes used to justify clubs and competitions (6, 7).

Treating self-organized physical activity only as a means to other ends overlooks a distinction that policy itself recognizes. Norwegian policy, for example, separates the intrinsic value (egenverdi) of sport and physical activity, the worth of the activity in itself, from its utility value (nytteverdi), the downstream effects that justify public spending (8). The review uses intrinsic value (egenverdi) in this policy and ordinary-language sense, the worth the activity has as an end in itself, as it is lived. In the philosophical taxonomy, this is final, non-instrumental value rather than intrinsic value in the strict sense of value grounded in an object's non-relational properties (9). In practice, measurement and funding follow the instrumental side, so intrinsic value is acknowledged in principle but left under-developed in the instruments that shape provision. This review takes that intrinsic value as its starting point. Rather than asking what self-organized physical activity yields, it asks what is distinctively valuable about physical activity being self-organized. Its central claim is that, for self-organized physical activity, intrinsic value is bound up with the self-organizing itself.

A growing body of scholarship has begun to take self-organized physical activity seriously on its own terms. Studies of lifestyle and informal sport have documented how these forms generate distinctive identities, subcultures, and ways of participating (7, 10, 11), and research on self-organized youth sport has shown that freedom from external control is central to how young people experience it (12). Two recent reviews have consolidated much of this literature. Säfvenbom et al. (13) synthesized the developmental outcomes associated with informal and lifestyle sport, linking participation to a range of positive developmental and psychosocial gains, while Andersson (14) examined the spatial conditions that self-organized sport depends on, showing how its value is tied to the places in which it unfolds. Together, these reviews establish that self-organized physical activity is both developmentally consequential and bound to the settings in which it takes place. This review extends that work by examining what makes self-organized physical activity valuable in itself, a question the two reviews do not take up directly. It draws together research on physical activity and self-determination theory, the sociology of sport, and the philosophy of sport.

The aim of this narrative review is to provide an account of the intrinsic value of self-organized sport and physical activity and of why this form of activity is significant. It is organized around three questions: what is distinctively valuable about physical activity being self-organized, why this form of activity is significant beyond the individual, and what the synthesis implies for how the activity is provided for and studied. In addressing them, the review describes how the self-organized character supports the qualities that give the activity its value, considers the significance of those qualities for participation, examines the conditions under which they are supported or eroded, and weighs the evidence that qualifies the account. In doing so, it treats the difference between organized and self-organized participation as a continuum of formalization, not a binary (11), and it distinguishes three broad types of self-organized physical activity, self-directed, scene-based, and semi-organized, which are introduced where the source of the activity's value is examined.

## Search strategy and study selection

This narrative review is based on a structured search of the published literature carried out in June 2026. The search used explicit Boolean strings in two databases chosen to span the relevant fields: PubMed (MEDLINE) for the physical-activity, exercise-psychology, and self-determination-theory literature, and Scopus for the broader sport-studies, sociology-of-sport, leisure, and policy literature, including the main journals in which research on self-organized and lifestyle sport appears. The reference lists of the most relevant reviews and articles were then screened for further relevant sources. The contents of journals that publish frequently on self-organized and lifestyle sport were also hand-searched. The approach followed published guidance for narrative reviews (15, 16), adopting elements of systematic searching to limit selection bias. A narrative design suits the aim of this review, which is a conceptual synthesis across several fields with different methods and vocabularies: physical-activity science and self-determination theory, the sociology of sport, and the philosophy of sport. No single systematic protocol spans these fields, and for syntheses of this kind, the criteria used to appraise narrative reviews emphasize a transparent search and a justified basis for selection over an exhaustive, reproducible screen (17).

Each search combined two blocks of terms with the Boolean operator AND: one for the activity forms of interest (self-organized, informal, lifestyle, action, and recreational physical activity and sport, and specific activities such as parkour, skateboarding, and climbing) and one for the experiential and intrinsic-value constructs of interest (intrinsic motivation, self-determination, autonomy, enjoyment, identity, belonging, meaning, and autotelic activity). The full strings for PubMed and Scopus are given in Table 1. Database searches were limited to English-language records published between 2000 and 2026; seminal theoretical works predating this window, such as foundational statements of self-determination theory and social identity theory, were retained as background references.

**Table 1:** Search strings by database.

#	Source	Search string	Records
1	PubMed	(parkour OR skateboard* OR surfing OR climbing OR “lifestyle sport” OR “lifestyle sports” OR “action sport” OR “action sports” OR “informal sport” OR “informal sports”) AND (identity OR “self-expression” OR subculture OR “self-concept”)	144
2	PubMed	(“informal sport” OR “informal sports” OR “self-organized” OR “self-organised” OR “lifestyle sport” OR “lifestyle sports” OR “street sport” OR “street sports”) AND (belonging OR inclusion OR community OR “social capital”)	284
3	PubMed	(“self-organized” OR “self-organised” OR informal OR “lifestyle sport” OR “lifestyle sports” OR “action sport” OR “action sports” OR parkour OR skateboard* OR “recreational physical activity” OR “recreational physical activities”) AND (“self-determination” OR “intrinsic motivation” OR autonomy)	17,750
4	PubMed	(“physical activity” OR exercise OR sport) AND (enjoyment OR “positive affect”) AND (adherence OR maintenance OR persistence)	1,077
5	PubMed	(“youth sport” OR “sport participation” OR “organized sport”) AND (“social inclusion” OR “social exclusion” OR inequality) AND (structural OR ethnicity OR disadvantage)	19
6	PubMed	(“intrinsic value” OR autotelic OR “for its own sake”) AND (sport OR “physical activity” OR play)	56
7	Scopus	(“self-determination theory” OR “intrinsic motivation”) AND (“lifestyle sport*” OR “action sport*” OR “informal sport*” OR skateboarding OR “rock climbing” OR surfing OR snowboarding)	20
8	Scopus	(“lifestyle sport*” OR “action sport*” OR “informal sport*”) AND (identity OR subculture OR belonging OR community OR culture)	228
9	Scopus	(“informal sport*” OR “self-organized sport*” OR “self-organised sport*” OR “lifestyle sport*” OR “street sport*”) AND (belonging OR “social inclusion” OR community OR integration OR loneliness)	108

Searches run June 2026; English language; 2000–2026. In PubMed, truncation (*) is not supported within quoted phrases, so phrase variants are written out explicitly; Scopus supports truncation within double-quoted phrases. The final column gives the search yield for each string (June 2026; publication years 2000–2026), as verified by re-running the strings as printed on 1 July 2026; the large count for string 3 reflects PubMed stemming of the unquoted term informal to information, so raw yields overstate the on-topic literature. Consistent with a narrative design, records were assessed for relevance and selected purposively, together with reference-list screening and hand-searching, and the synthesis draws on the 63 sources listed in the References.

The studies were eligible if they concerned (a) self-organized, informal, lifestyle, or otherwise participant-governed sport or physical activity, or physical activity in general where a construct of interest required the broader literature, and (b) at least one of the experiential constructs named above. Conceptual and empirical work was eligible, including qualitative and quantitative studies, systematic reviews, and meta-analyses; records concerning elite or high-performance sport, clinical exercise interventions, or physical activity undertaken solely for transport or occupation were excluded. In practice, records returned by each string were screened on title and abstract against these criteria, the largest yields in relevance-ranked order, and the full text was retrieved and read wherever title and abstract left eligibility unclear. The selected literature was synthesized thematically. The synthesis is interpretive, reading the literature through the lens of intrinsic value and self-determination theory, so it is selective rather than exhaustive, and other readers might weight the literature differently. Throughout, the review distinguishes evidence that examines self-organized or informal physical activity directly from evidence used more indirectly, such as evidence from lifestyle and adventure sport or from physical activity in general, and from theoretical work in self-determination theory, identity theory, and the philosophy of sport; this gradation is made explicit in Table 2. In this grading, directness follows the setting studied and not the activity label: evidence from lifestyle sport counts as direct where the participation studied is informal and participant-governed, and as more indirect where it concerns organized or commercial provision of such activities, or where the literature does not separate the two.

**Table 2:** Summary of the evidence on the intrinsic value of self-organized sport and physical activity, by experiential dimension.

Dimension	What self-organization affords	Representative sources	Evidence type	Strength and scope
Autonomy	Volition and self-direction; the activity is defined on the participant's own terms	Ryan and Deci (21); Teixeira et al. (24); Xu et al. (25); Lee et al. (29); Mangan et al. (30); Houge Mackenzie and Hodge (34)	Theory; systematic and umbrella reviews; small empirical studies (directness: mostly indirect and theoretical)	The motivation–adherence link is well supported for physical activity in general; for lifestyle and adventure sport, review-level syntheses now complement the smaller primary studies
Mastery on one's own terms	Competence pursued at a self-set pace, without external selection	Säfvenbom et al. (13); Babic et al. (35); Lubans et al. (36); Brussoni et al. (37)	Scoping and systematic reviews; meta-analysis (directness: mostly indirect)	Self-concept and self-esteem links are strong but concern physical activity broadly; self-organized specificity is review-level
Self-expression and identity	Identity and subcultural belonging shaped by participants themselves	Wheaton (10); Säfvenbom et al. (13); Andersson (14); Skille and Skille (41); Glenney et al. (44); Ellmer et al. (47)	Foundational monograph; reviews; qualitative studies (directness: direct)	Rich qualitative and cultural evidence across activities and settings
Meaning and experience	Lived, autotelic experience valued for its own sake	Reimer (18); Clegg and Butryn (31); Taylor and Carr (32); Boudreau et al. (20); Martinho et al. (33)	Philosophy; conceptual and qualitative studies; systematic and scoping reviews (directness: theoretical and interpretive)	Conceptual and qualitative, now supported by review-level syntheses of flow and experience; intrinsic value is interpreted rather than measured directly
Belonging	Community formed around shared interest rather than membership	Säfvenbom et al. (13); Warner et al. (48); Spaaij et al. (49); Ogundipe et al. (50); Book (51)	Reviews; comparative and qualitative studies (directness: direct)	Consistent across studies, though access is patterned by social position (52)

## The self-organizing as the source of value

The premise of this review is that what gives self-organized physical activity its distinctive worth is the self-organizing. The value does not arise in spite of the absence of coaches, federations, and fixed competitive formats, but because participants themselves govern the content, intensity, timing, style, and meaning of what they do. Intrinsic value, in this sense, is an evaluative notion: it refers to the worth an activity has as it is lived, independent of any product it yields. The experienced qualities set out below are constituents of this value rather than causes of a separate good: the worth lies in their being lived, not in any outcome they may later produce. The distinction can be drawn for each quality in turn. Autonomy may be valued as an outcome, in that taking part can strengthen a person's longer-term sense of agency; but it is valued here as a constituent of the activity's worth, because the participant is already acting on self-chosen terms while taking part. Belonging, identity, and meaning admit the same distinction: they may also follow from participation as developmental or psychosocial outcomes, yet they are part of the value of the activity as it is lived, namely the belonging felt in the doing, the identity expressed in it, and the meaning made through it. It is this experienced, non-derivative aspect, not any later effect, that gives the activity its value as an end in itself (9). In the philosophy of sport and play this is the autotelic quality of an activity, something done for its own sake; the idea has recently been defended as central to the value of play (18), while others have examined the difficulty of specifying precisely when an activity is pursued autotelically rather than for mixed reasons (19). Intrinsic value should be distinguished from intrinsic motivation, a psychological construct concerning why people act. The two are connected, in that evidence of intrinsic motivation indicates that participants treat an activity as worth doing in itself, but the value claim is not reducible to the motivational one. The self-organized form is a setting in which the autotelic quality is readily realized, because the activity is defined on the participant's own terms. This premise is anchored in the literature synthesized below, in which participants across informal and lifestyle sport describe the activity as valued in the doing itself, for its freedom, absorption, and fellowship (12, 13, 20). Figure 1 summarizes the argument developed in the sections that follow.

**Figure 1:**
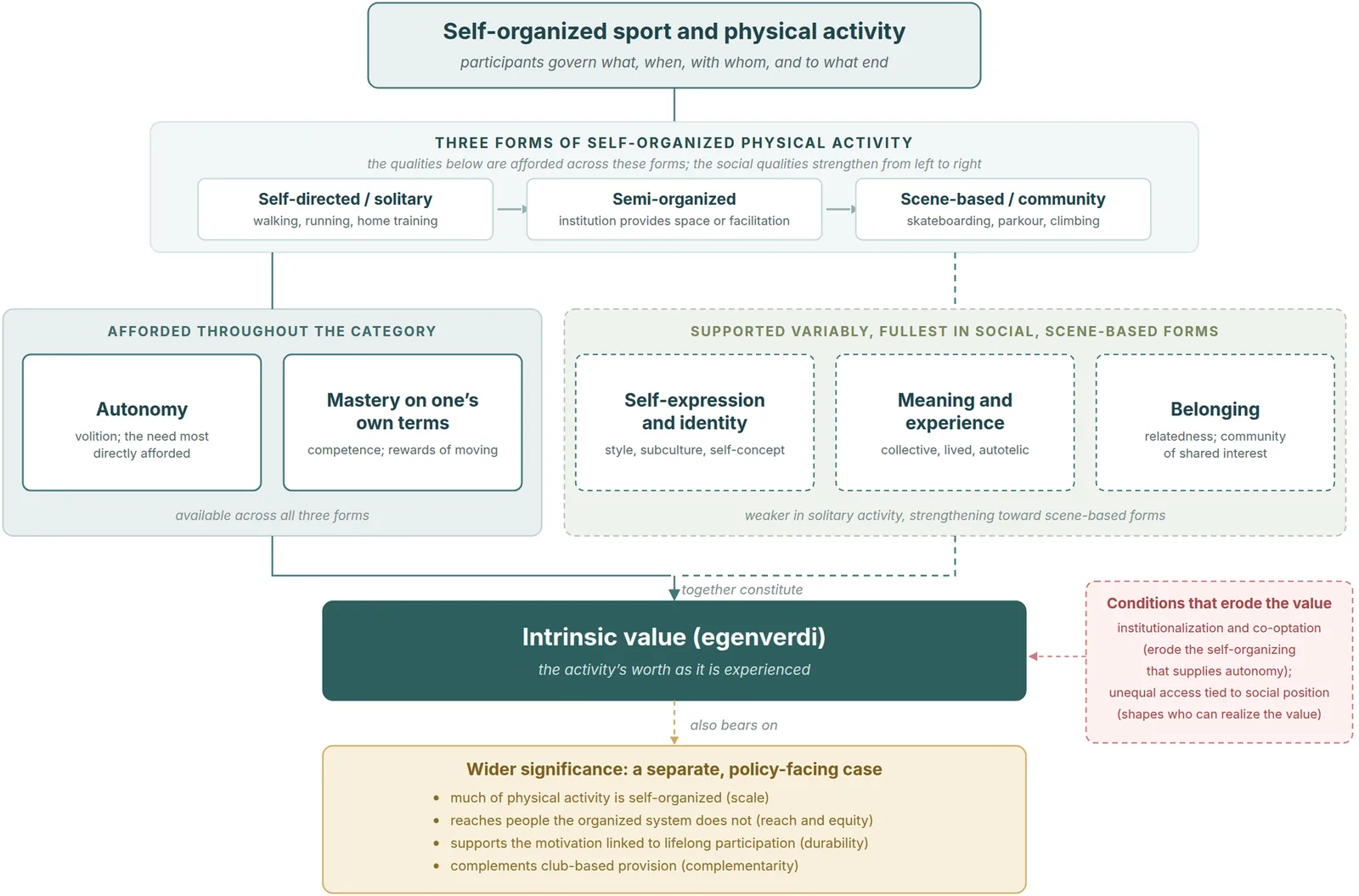
Conceptual framework of the review. Self-organized sport and physical activity, in which participants govern what they do, when, with whom, and to what end, spans three broad forms: self-directed individual activity, semi-organized activity in which an institution provides space or facilitation while participants retain substantial capacity to negotiate the terms, and scene-based community activity. The qualities the activity affords are distributed unevenly across these forms. Autonomy and mastery on one's own terms, including the immediate rewards of moving, are available across the whole category (solid outlines and connectors), while self-expression and identity, meaning and experience, and belonging are supported more variably and are realized most fully in the social, scene-based forms (dashed outlines and connectors). Together these experienced qualities constitute the intrinsic value (egenverdi) of the activity, its worth as it is experienced. Institutionalization and co-optation can erode the self-organizing that supplies autonomy, and unequal access tied to social position shapes who can realize the value. The activity's wider significance for participation and provision is a separate, policy-facing consideration, not the purpose of the value.

Self-determination theory helps explain why the self-organizing matters for motivation and well-being. The theory holds that motivation is most autonomous, and well-being best supported, when three basic needs are met: autonomy, a sense of volition; competence, a sense of effectiveness and mastery; and relatedness, a sense of connection (21). Of these, autonomy is the one most directly afforded by self-organization, since competence pursued under one's own direction and relatedness formed around a chosen activity differ from competence and connection imposed from outside. The three basic needs are co-equal and separately essential; autonomy is simply the one self-organization most directly engages, not a need that governs the others, and even this is an affordance rather than a guarantee, since the structural freedom to choose and experienced volition can come apart (22, 23). The point is not that self-organization monopolizes these satisfactions: autonomy can also be supported in organized settings through autonomy-supportive coaching, and self-organization does not by itself guarantee competence or relatedness, since a person exercising alone may experience little of the latter. The claim is the more limited one that self-organized forms structurally favor autonomy. In the context of movement, autonomous and intrinsic forms of motivation are the kinds most consistently associated with sustained physical activity. A systematic review of exercise and self-determination theory found autonomous motivation and intrinsic regulation to be reliably associated with adherence (24), and a review of systematic reviews reached the same conclusion across age groups, recommending that interventions support perceived competence and autonomous motivation rather than rely on external pressure or reward (25).

The terminology here is varied: self-organized, informal, lifestyle, alternative, and action sport are used in overlapping ways, but a common thread is that these activities are not unorganized so much as organized by the participants themselves, with structure emerging from their interaction rather than from formal rules and hierarchies (12, 26). O’Connor et al. (11) argue that a binary of formal and informal sport is too coarse, and that participation is better understood as a continuum of formalization, defined by how far participants can negotiate the terms of the activity. Gilchrist and Wheaton (7) place informal and lifestyle sport at the edges of established structures, with distinctive forms of organization and belonging. The value at stake is therefore a matter of degree, and it is greatest where participant governance is greatest. Here, self-organized serves as an umbrella term, while individual studies use narrower labels; where evidence concerns children's free play, it is treated as related to, rather than identical with, adult self-organized physical activity. The category is also internally heterogeneous. It spans solitary, self-directed exercise such as walking, running, and home training, as well as social, scene-based forms such as skateboarding, parkour, climbing, and street sports, and the qualities examined below apply unevenly across this span. Autonomy and the immediate rewards of moving are available throughout, whereas identity, subculture, and belonging are documented chiefly in the scene-based forms; the synthesis notes where the evidence is confined to them. Within this span, and along the continuum of formalization noted above, it is useful to distinguish three broad types that differ in their social organization and in the qualities they most strongly afford. Self-directed individual activities, such as walking, running, and home training, turn chiefly on autonomy, flexibility, and the immediate enjoyment of moving (24, 27). Scene-based, community activities, such as skateboarding, parkour, climbing, and street football, more strongly develop identity, subculture, belonging, and collective meaning (7, 10, 13). Semi-organized activities, in which an institution provides space, equipment, or facilitation while participants retain substantial capacity to negotiate the terms of participation (11), sit between the two; the organized parkour classes studied by Carson et al. (28) are one example. The account that follows applies most fully to the scene-based type, and the qualities it identifies are afforded unevenly across the three.

## What self-organization affords

The qualities that self-organization affords can be grouped under the basic needs it satisfies; Table 2 summarizes the evidence by dimension. The strength and directness of that evidence vary, and Table 2 records this: the evidence for self-expression, identity, and belonging is largely direct, drawn from studies of informal and lifestyle sport; the evidence for autonomy and mastery is more often indirect, resting on the broader physical-activity, self-determination, and adventure-recreation literatures; and the account of meaning and autotelic value is chiefly theoretical and interpretive. Strength of affording and directness of evidence are, moreover, distinct: autonomy is the quality most directly afforded by self-organization yet rests largely on indirect evidence, whereas identity and belonging, afforded chiefly in the scene-based forms, are supported by more direct evidence. The first is autonomy together with mastery on one's own terms. Studies that ask participants in lifestyle sport why they take part return repeatedly to choice and self-direction. In an exploratory study of 27 children and adolescents enrolled in organized parkour classes, a noncompetitive lifestyle sport, Carson et al. (28) recorded high scores on measures of interest and enjoyment and of perceived choice, and their interviews emphasized autonomy and enjoyment alongside camaraderie, creativity, and a self-directed wish to improve. Because the setting was organized provision of a lifestyle sport rather than self-organized physical activity, the study bears on the continuum point above, that the content and ethos of these activities can support autonomy even in formalized formats, rather than on self-organization as such; the sample is also small, so the findings are indicative. A study of climbers similarly found that more self-determined forms of motivation accompanied deeper and more sustained involvement in the activity (29), and a systematic review of the psychology of rock climbing identifies intrinsic motivation, task orientation, and flow as recurrent features of the sport (30). Alongside choice, these studies return to the experience of the activity itself, an absorption practitioners value in the doing, not for anything it yields. A phenomenological study of parkour and freerunning likewise foregrounded the lived experience of the activity, its play, movement, and risk, as central to why practitioners take part (31), and mountain bikers describe a search for flow and “living in the moment” in similar terms (32). These accounts are borne out at review level: a systematic review and thematic synthesis found flow, the absorbing experience often valued as an end in itself, to be a central and recurring feature of adventure recreation across activities such as climbing, surfing, and kayaking (20), and a scoping review of extreme sport participants synthesizes the motives and experiential qualities that draw people to them (33). A conceptual framework connects the autonomy, competence, and relatedness afforded by self-directed adventure recreation to subjective well-being and a sense of flourishing (34). A scoping review of informal and lifestyle sport points the same way, reporting that across the studies it synthesizes, young people describe self-organized physical activity as a source of freedom, enjoyment, and self-determination (13). Taken together, primary studies and reviews converge, across methods and across activities as different as climbing, parkour, and mountain biking, on choice and self-direction as central to why participants value these activities. Mastery follows the same logic: the review reports self-efficacy and self-improvement as recurring outcomes, and self-organized forms can leave particular room for these among those who did not find a place in competitive sport (13). The general literature is consistent, with physical activity associated with physical self-concept and perceived competence and, in turn, with global self-esteem in young people (35, 36), and with associations between outdoor play and self-esteem in children (37). The immediate enjoyment of moving belongs here too: physical activity is associated with positive emotions, particularly in children and young people (38), and more autonomous, enjoyment-based motivation is associated with continued participation (24), a pattern that recurs across the life course, including among older adults for whom outdoor, self-directed activity supports intrinsic motivation (27).

The second quality is self-expression and identity. Because participants shape the form, style, and meaning of the activity, self-organized settings give particular room for individual identity work (13), consistent with the role of group membership in constituting identity (39) and with identity formation as a developmental task that continues across life (40). Lifestyle sport has long been analyzed as a domain of consumption, identity, and difference, in which participants construct distinctive identities and forms of subcultural belonging (10). Practitioners develop subcultures around activities that are typically self-organized, and for many the activity becomes part of who they are (13), with young people combining elements to develop their own style (14). Recent work in Norway shows backcountry skiers making meaning through their sensuous engagement with nature, positioning the activity between a global lifestyle sport and a national tradition (41). This has a measurable counterpart in adulthood, where exercise identity is associated with exercise behavior (42, 43). Self-organized physical activity also reinterprets ordinary space as a field for expression, as in accounts of urban skateboarding (44), and it serves as a setting for learning in which young people develop skills and leadership by negotiating roles and rules for themselves (45, 46), the latter examining these processes where informal sport is brought into physical education through negotiated practice; a scoping review of learning in action sports found such learning to be largely informal and self-regulated, organized by participants rather than directed from outside (47).

The third quality is belonging. Self-organized settings form community around shared interest rather than formal membership, which lowers the threshold for those who do not fit, or do not wish to fit, conventional club structures (13). Empirical studies bear this pattern out across quite different settings and populations. A focus-group study comparing formal and informal sport found that the two build a sense of community in different ways, drawing on factors such as common interest, social spaces, and voluntary action as well as administration and competition (48). An Australian study of informal sport describes participants, many with migration backgrounds, experiencing safe spaces in which they built networks and in some cases maintained language and culture, in contrast to other settings where they had felt unwelcome (49). Street football has been described as a means of social inclusion and recovery, with the team and setting becoming identity-bearing (50), and a self-organized scene can become a community with which a wider public identifies (51).

## The significance of self-organized physical activity

The significance of these qualities extends beyond the individual participant. The argument here is deliberately two-level: the preceding sections ground the case for self-organized physical activity in its intrinsic value, while the considerations that follow are prudential, addressed to a policy field that allocates resources on instrumental grounds. They do not replace the intrinsic case but show that taking it seriously is also consistent with that field's own aims. Four considerations drawn from the literature indicate why.

First, scale. In many populations most physical activity takes place outside organized club sport, and much of it is self-organized, so a field organized around club participation addresses only a part of how people are actually active. European survey data support this. In the most recent Europe-wide survey, among those who are active, the settings reported most often are informal ones, parks and other outdoor spaces and the home, while sports clubs and fitness centers are each reported by a minority (3). These data describe where people are active rather than how the activity is organized, and some physical activity in informal settings is itself organized, as when a club session takes place in a park; they nonetheless indicate how much movement lies outside dedicated sport structures. Analyses of the dominant pyramid model of European sport make the same point: it assumes participation in grassroots clubs, while much physical activity takes place outside that structure (4).

Second, reach and equity. Self-organized physical activity tends to be low threshold, typically without membership, fixed times, or competitive selection, and it can be adapted to participants’ needs, which can allow it to include people whom organized structures do not (13, 53). A systematic review found that practical barriers such as time, cost, transport, and location limit children's sport participation (54), and self-organized forms, which require less of each, can lower these thresholds. This is significant given that participation in organized sport tends to fall through adolescence, with withdrawal often associated with reduced enjoyment, competitive pressure, or a sense of not being good enough (26, 55). Self-organized physical activity offers a different basis for participation, in which competence is cultivated on the participant's own terms.

Third, durability. The autonomy and enjoyment that self-organization affords are the conditions most consistently associated with sustained participation (24, 25), and the pattern holds across the life course (27). Within this evidence the relationship is not carried by intrinsic motivation alone: identified regulation, an autonomous but outcome-oriented form of motivation, tends to predict initial adoption more strongly, while intrinsic motivation is the better predictor of long-term adherence (24). An activity experienced as worth doing for its own sake is more likely to be continued, which is also the precondition for whatever instrumental benefits follow. Empirical work across two European countries indicates that fostering unorganized sport can help sustain adolescent participation (56). Studies of repeated participation in organized endurance events offer a contrasting case; they fall outside the review's eligibility criteria and are cited for comparative context only, not as part of the synthesis. In these settings, the intention to return is explained by attachment to the particular event, the quality of the event environment, and health motives (57), although participants’ standing involvement with the activity itself outweighs the attractiveness of the event (58). The contrast underscores the argument of this paragraph: repeated participation in organized events leans on qualities of the provision, while the durability of self-organized physical activity rests on the autonomous and intrinsic motivation that the activity itself affords.

Fourth, complementarity. Prioritizing self-organized physical activity does not mean displacing organized sport. On instrumental measures the comparative evidence, if anything, favors the organized form: in a national study of Norwegian adolescents, participation in organized sport showed stronger associations with developmental factors than self-organized physical activity, while taking part in both was associated with more favorable profiles than taking part in only one (26), and a systematic review of children's and adolescents’ sport suggests that sport, particularly team sport, may be associated with psychosocial benefits beyond those attributable to physical activity in general (59). These comparisons concern instrumental outcomes rather than the experienced qualities at issue here, and the combination finding points toward a fuller portfolio, in which the structure of organized sport and the autonomy of self-organized physical activity each have a place.

## Implications for provision, policy, and research

If much of how people are active is self-organized, and this activity reaches the underserved, supports lifelong participation, and complements organized sport, the question is why it receives comparatively little dedicated support. Part of the explanation is that the instruments of the field are calibrated to organized structures and to measurable outcomes. Funding follows membership-based organizations and competitive pathways; facility planning is shaped by the requirements of formal sport; and measurement records participation in countable units, which favors the organized and renders the self-organized close to invisible. Treated as a residual, self-organized physical activity is rarely the explicit object of a policy instrument, and so the value described above is left unsupported by default rather than by decision. Research on the management of informal sport reaches a similar conclusion: stakeholders responsible for community sport report that existing practices, designed around organized provision, limit the potential of informal sport (60). The spontaneous, self-organized use of public space has likewise been described as an under-recognized promoter of both public health and sport participation (6). The same pattern is visible at the European level. Analyses of the European Sport Model argue that its club-and-federation pyramid does not capture how most Europeans organize and engage in physical activity (4, 5), and although European institutions have begun to recognize self-organized sport as a significant share of physical activity that is distinct from organized sport, this recognition has not yet been matched by instruments designed for it (5).

Several implications for provision and policy follow from the synthesis. Because the value of self-organized physical activity rests on autonomy, the most useful support is the kind that preserves it. The Malmö case illustrates both the promise and the risk: municipal engagement can sustain a self-organized scene, but a professionalized model for representing participants’ interests can also erode the grassroots involvement and self-determination that gave the scene its meaning (51). Studies of the institutionalization of parkour reach a similar conclusion, that drawing self-organized physical activity into formal sport structures can blunt what makes it distinctive (61). This points to provision that is autonomy-preserving rather than provision that formalizes the activity into the image of organized sport.

Two further implications follow. The first concerns the conditions of use. Equal facilities do not produce equal use, and whether a space delivers the value described here depends on how it is run, on hosting, maintenance, lighting, winter operation, and accessibility in the broad sense, as much as on the capital cost of building it. These measures are often modest relative to new facilities, yet they tend to fall outside the categories that funding instruments recognize, which is where additional attention could matter most. Questions of spatial justice, of who can reach and use a given space, also shape whether its value is realized (62). The second concerns knowledge. If the value of self-organized physical activity is to inform decisions, it has to be made visible, which means treating self-organized and semi-organized physical activity as a primary object of mapping and measurement, and complementing outcome measures with measures of the experience of physical activity, including autonomy, enjoyment, identity, and belonging. On this account, supporting self-organized physical activity is less a matter of large new programs than of directing attention and modest resources toward where most movement actually happens and the conditions that sustain it. These principles translate into concrete measures. In provision and planning, it means designing and programming public space for self-organized use (skate spots and parkour features, ball walls and street-sport surfaces, outdoor gyms, and lit, maintained, winter-operated areas open to informal physical activity), not only building facilities for organized sport (6, 62). In funding, it means instruments that can reach activities without a membership-based club to apply on their behalf, such as area- or place-based grants, support routed through municipalities or open to loose collectives, and support for hosting and maintenance, not only capital construction (60). In measurement, it means complementing counts of club membership with data on where and how people are actually active and with measures of the experiential qualities identified here (autonomy, enjoyment, identity, and belonging). In governance, it means including participants in decisions about the spaces and scenes they use without professionalizing or bureaucratizing those scenes, since formal representation can itself erode the self-determination it is meant to serve (51). And because access to self-organized physical activity is patterned by social position, including gender (63), migration background (49), and class (52), and likely also disability and age, each measure should be assessed for whether it widens or narrows that gap, not assumed to reach everyone equally.

## Tensions and limitations

Several lines of evidence qualify the argument. The first concerns the direction of the relationship between participation and inclusion. A qualitative longitudinal study of young people in Norway found that organized sport participation tends to reflect existing social inclusion and security rather than to create it, with participants drawn disproportionately from those who already feel secure, and with the difficulties of non-participants tied to structural conditions of class and ethnicity (52). The finding concerns organized sport, but it cautions against assuming that the value of self-organized physical activity is equally available to all; access to autonomy, mastery, and belonging through movement is itself shaped by social position. The second concerns the limits of support already noted: institutional engagement that formalizes self-organized physical activity too far can undermine the autonomy that is its source of value (51), and a nominally open setting can still be exclusionary: street skateboarding, for instance, can develop gendered hierarchies that advantage some participants over others (63). The value of self-organized physical activity is conditional on the quality and character of provision, not only on its presence.

A final limitation concerns the evidence base and the scope of the review. Much of the most directly relevant work is qualitative, drawn from single studies, or theoretical, and several of the stronger quantitative syntheses concern physical activity in general rather than self-organized forms specifically.

## Conclusion

In many populations a large share of physical activity is self-organized, and its distinctive worth lies in the self-organizing itself, in the autonomy of acting on one's own terms, the mastery pursued without external selection, the self-expression and identity it allows, the meaning made through self-direction, and the belonging built around a shared pursuit. These qualities are unevenly distributed across the category: autonomy and enjoyment run throughout it, while identity and belonging are most fully realized in its social, scene-based forms. The value is therefore conditional as well as distinctive: self-organization affords these qualities without guaranteeing them, and their realization depends on autonomy-preserving, participant-governed conditions, on the social relations of the setting, and on who can reach and use it. This worth is not confined to the individual. Self-organized physical activity reaches people the organized system does not, supports the motivation associated with lifelong participation, and is sustained by relatively modest, well-targeted provision. A field whose instruments are geared to organized structures and measurable outcomes is apt to treat it as a residual. The synthesis indicates that recognizing the intrinsic value of self-organized physical activity has implications for how it is conceptualized, provided for, and studied, including autonomy-preserving provision, attention to the conditions of use, and knowledge that makes the activity visible.
